# Metamizole in the Management of Musculoskeletal Disorders: Current Concept Review

**DOI:** 10.3390/jcm13164794

**Published:** 2024-08-14

**Authors:** Naveen Jeyaraman, Filippo Migliorini, Shrideavi Murugan, Swaminathan Ramasubramanian, Sangeetha Balaji, Nicola Maffulli, Madhan Jeyaraman

**Affiliations:** 1 Department of Orthopaedics, ACS Medical College and Hospital, Dr MGR Educational and Research Institute, Chennai 600077, Tamil Nadu, India; 2 Department of Orthopaedic and Trauma Surgery, Academic Hospital of Bolzano (SABES-ASDAA), 39100 Bolzano, Italy; 3 Department of Life Sciences, Health, and Health Professions, Link Campus University, 00165 Rome, Italy; 4 Department of Orthopaedics, Government Tirunelveli Medical College and Hospital, Tirunelveli 627002, Tamil Nadu, India; 5 Department of Orthopaedics, Government Medical College, Omandurar Government Estate, Chennai 600002, Tamil Nadu, India; 6 Department of Trauma and Orthopaedic Surgery, Faculty of Medicine and Psychology, University La Sapienza, 00185 Roma, Italy; 7 School of Pharmacy and Bioengineering, Keele University Faculty of Medicine, Stoke on Trent ST4 7QB, UK; 8 Centre for Sports and Exercise Medicine, Barts and the London School of Medicine and Dentistry, Mile End Hospital, Queen Mary University of London, London E1 4DG, UK

**Keywords:** metamizole, pyrazolone derivatives, musculoskeletal disorders, analgesia, pharmacokinetics, efficacy, safety

## Abstract

Metamizole, or dipyrone, has been used for decades as a non-narcotic analgesic, providing pain relief from musculoskeletal disorders and antipyretic and antispasmolytic properties. Despite being in use since the 1920s, its mechanism of action still needs to be discovered. Despite causing fewer adverse effects when compared to other analgesics, its harmful effects on the blood and lack of evidence regarding its teratogenicity make the usage of the drug questionable, which has led to it being removed from the drug market of various countries. This narrative review aims to provide a detailed insight into the mechanism of action and efficacy, comparing its effectiveness and safety with other classes of drugs and the safety profile of metamizole.

## 1. Introduction

Low back pain (LBP) continues to be the primary contributor to years lived with disability worldwide. In 2020, there were over 500 million prevalent cases of LBP globally. Although age-standardised rates have slightly declined over the past three decades, the projected scenario for 2050 is concerning because more than 800 million people are expected to suffer from LBP [1]. Musculoskeletal disorders (MSDs), including neck pain, were ranked subsequently at third and fourth positions, respectively [2]. MSDs, as per the definition of the World Health Organization, include health issues affecting the musculoskeletal system. MSDs include a broad spectrum ranging from slight discomfort to incapacitating injuries [3]. Sprains, strains, tears, soreness, pain, hernias, carpal tunnel syndrome, and connective tissue injuries are prevalent MSDs [4]. Musculoskeletal pain has a significant impact on the quality of life [5]. Chronic musculoskeletal pain disrupts daily life through sleep disruption, depressed mood, fatigue, and various other limitations [5].

Globally, MSDs are on the rise, posing significant challenges in interventions and resource allocation [6]. Although MSDs impact various age groups, their prevalence notably rises with age. In those over 65, around three out of four individuals suffer from MSDs [7]. From 1996–1998 to 2009–2011, the incidence of MSDs surged by 19%, reaching 102.5 million cases [8]. Economic burdens in the same period skyrocketed from USD 367.1 billion to USD 796.3 billion, a 117% increase. In lower middle-income countries (Tanzania), the rise in those with MSDs proves to be a burden, because over 10% of the income is allocated to treating the disorders, which is often 2–3 times the usual medical expense [9]. With over 88.8% of the patients earning less than USD 250, 73.7% stated that healthcare expenses were a catastrophic burden [10]. Economic burden (for RA) in direct costs (mainly drugs, comprising about 87% of the expenditure) range from USD 401 to USD 67,306, and indirect costs such as absenteeism and work disability account for 39% to 86% of the costs, ranging from USD 509 to USD 22,444 [11].

Additionally, post-injury disability led to 47% of patients being unable to return to full employment [12]. Furthermore, patients also reported a decrease in individual and familial wages in 88% and 33% of injuries, respectively, and intra-family labour reallocation in 83% of the injuries [13,14]. Non-pharmacological approaches such as self-management, education, exercise, and psychosocial interventions play a pivotal role in pain management [4]. Exercise and psychosocial modalities, particularly aerobic and resistance exercises, show benefits [15,16]. Complementary treatments, such as acupuncture, can be effective [17]. Pharmacological interventions, which include analgesics and corticosteroid injections, aim for short-term relief [16,18]. NSAIDs and opioids, although showing modest effects, carry risks for potential adverse effects such as sedation and mood swings [16]. Photo biomodulation therapy, using light, has emerged as a novel approach to pain management [18].

Metamizole, a pyrazolone derivative and non-opioid analgesic with the N02BB02 Anatomical Therapeutic Chemical code, has been used since 1922 primarily for pain relief, fever reduction, and antispasmodic effects [19,20,21]. The chemical formula of the drug is N-(2,3-dimethyl-5-oxo-1-phenyl-3-pyrazolin-4-yl) [22]. Various studies support its efficacy in managing pain with different origins and intensities, such as postoperative pain, cancer pain, headaches, and migraines. Notably, it demonstrates safety in short-term use with adverse effects on the lower gastrointestinal, cardiovascular, renal, and neurological systems [19]. Initially considered a non-steroidal anti-inflammatory drug, metamizole was later reclassified as a non-narcotic analgesic given its inhibitory action on central cyclooxygenase(COX)-3 [22]. There are numerous reviews stating the efficacy of the drug, often claiming it to be similar or superior to NSAIDs in some instances [23,24]. Although concerns have been previously raised on agranulocytosis caused by the drug, a previous study [25] showed the incidence to be lesser than more commonly administered medications such as antithyroid drugs and ticlopidine. The event of mild adverse effects was also lesser in those taking metamizole when compared to other non-opioid analgesics [26]. Reviews have also previously been conducted on the efficacy of the drug when combined with other non-opioid analgesics. However, it is to be noted that all previous reviews focused singularly on one particular aspect of the drug [27,28], and no review focused on the overall efficacy of the drug across various causes of musculoskeletal disorders.

In recent years, several comprehensive reviews have examined metamizole’s role in managing musculoskeletal disorders, providing valuable insights into its efficacy, safety, and pharmacological profile. These reviews have explored its pharmacological mechanisms, including its effects on COX-3 inhibition and potential interactions with cannabinoid and opioidergic systems. They have also confirmed its efficacy in various pain conditions, compared its effectiveness with other analgesics, and addressed ongoing safety concerns, particularly regarding agranulocytosis [29]. Despite these contributions, there remains a need for a holistic review that synthesises these findings across the spectrum of musculoskeletal disorders, considering both its therapeutic potential and safety profile in diverse clinical contexts.

The review aims to summarise the efficacy and safety profile of metamizole drugs in treating various musculoskeletal disorders. This review introduces the pharmacology of the drug, followed by clinical indications and evidence of the drug efficacy, safety profile, comparative analysis with other opioid and non-opioid analgesics, various challenges associated with current research on the drug, and concluding with possible unexplored aspects of the drug that may solidify its role in treating multiple demographics of the population.

## 2. Methodology

A comprehensive literature search was conducted using PubMed, MEDLINE, Cochrane Library, Scopus, and Web of Science to identify studies on the efficacy and safety of metamizole in managing musculoskeletal disorders. The search strategy included keywords and MeSH terms such as “Metamizole”, “Dipyrone”, “musculoskeletal disorders”, “analgesia”, “pain management”, “efficacy”, “safety”, and “adverse effects”. Inclusion criteria were studies involving metamizole for treating musculoskeletal disorders, published in English, involving human subjects, and including randomised controlled trials (RCTs), cohort studies, case-control studies, cross-sectional studies, and systematic reviews that reported on the efficacy, safety, and adverse effects of metamizole. Exclusion criteria were studies unrelated to metamizole, animal studies, articles not available in English, studies with insufficient data on outcomes, conference abstracts, editorials, and commentaries.

## 3. Pharmacology of Metamizole

Metamizole, represented by the chemical formula N-(2,3-dimethyl-5-oxo-1-phenyl-3-pyrazolin-4-yl), is a prodrug that spontaneously undergoes breakdown in an aqueous environment [22]. The drug is usually a white or a crystalline white powder that is very soluble in water and alcohol [30]. Pyrazolone derivatives, with their nearly neutral pKa value and minimal plasma protein binding, exhibit a homogeneous and rapid distribution throughout the body. This efficient dispersion is attributed to their exceptional ability to penetrate the blood–brain barrier easily [31]. Administered intravenously, the parent drug is detectable in the bloodstream for approximately 15 min, while oral administration results in its absence in plasma and urine [22]. Orally ingested, metamizole is non-enzymatically hydrolysed in gastric juice to produce 4-methyl-amino-antipyrine (MAA) with an 85% bioavailability. In the liver, cytochrome P450 (CYP) 3A4 further metabolises MAA into 4-formyl-amino-antipyrine (an end metabolite) and 4-amino-antipyrine (AA), the latter being acetylated into 4-acetyl-amino-antipyrine (AAA). AA and MAA transform into Arachidonoyl amides, penetrating the blood–brain barrier and reaching effective concentrations in the spinal cord to produce therapeutic effects [20,22]. Metamizole is commonly used to relieve visceral and somatic pain. Despite indications of its potential, it has not been employed for neuropathic pain [32].

**Figure 1:**
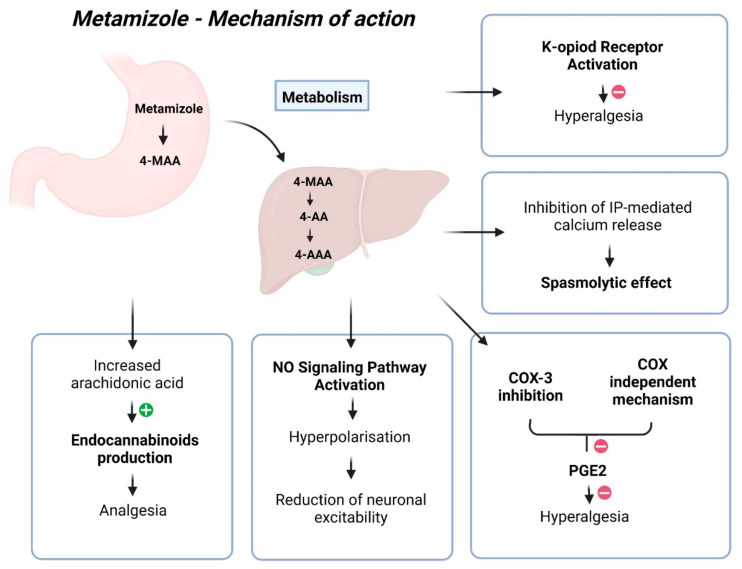
Mechanism of action of metamizole.

Despite more than 90 years of use, the mechanism of action for metamizole remains unclear, with its mechanism of analgesia being quite complex (Figure 1) [22]. The most probable explanation involves its impact on COX-3, the cannabinoid system, and the opioidergic system [22]. COX-3 retardation leads to reduced Prostaglandin E2 synthesis, diminishing nociceptor sensitivity and excitability, thereby achieving an analgesic effect [22]. However, the analgesic effect of metamizole is not associated with its capacity to inhibit prostaglandins [31]. The pharmacologically active metabolites of metamizole, MAA, and AA differ from classical COX inhibitors because they do not inhibit COX activity in vitro. Instead, they redirect prostaglandin synthesis, eliminating the possibility of COX inhibition through binding to its active site [33]. Many authors have emphasised the inhibition of COX activity as the primary mechanism of action for metamizole, with its action on COX-2 being tenfold more potent than COX-1 [34]. However, findings suggest that metamizole metabolites can directly impede hyperalgesia induced by prostaglandin E2 (PGE2) and isoprenaline through a COX-independent mechanism. Like paracetamol, metamizole exhibits COX inhibition activity in vitro, mainly targeting COX-2, yet demonstrates weak anti-inflammatory properties and minimal gastrointestinal toxicity in humans [35]. Reducing prostaglandin synthesis inhibits pain-related signals and increases the availability of arachidonic acid to produce endocannabinoids, which exert analgesic effects in the spinal cord [30]. Previous investigations have reported that metamizole inhibits prostaglandin formation, deduced by assessing PGF2a and two primary urinary metabolites of prostacyclin [36,37,38,39,40]. The pharmaceutical compound exhibits specific anti-inflammatory properties, albeit to a lesser extent when juxtaposed with alternative medications, potentially attributed to its diminished affinity for COX in environments characterised by elevated peroxide levels, such as inflamed tissues [21].

Other potential mechanisms include the agonistic effect of metamizole on type 1 CB1 cannabinoid receptors, reducing GABAergic transmission in the periaqueductal grey matter. This mainly disinhibits glutaminergic activating neurons, activating the descending pathway, resulting in antinociception. Additionally, the analgesic effect of metamizole may activate the endogenous opioidergic system [22]. The drug may also directly hinder nociceptor sensitisation by activating the NO signalling pathway. The authors hypothesised that metamizole facilitates activation of the PI3Kγ/AKT/nNOS/cGMP pathway, leading to the hyperpolarisation of primary sensory neuron terminals and a subsequent reduction in neuronal excitability [41]. Additional studies have found that the anti-hyperalgesic effect of 4-MAA relies on κ-opioid receptor activation, functioning similarly to a morphine-like drug [35]. Another likely mechanism involves the delayed initiation of the l-arginine/NO/cGMP/K^+^ channel pathway in the periphery and the spinal cord [30]. Metamizole induces the relaxation of G protein–coupled receptor-mediated contractions in isolated guinea pig tracheal smooth muscle. The observed metamizole-induced reduction in ATP-triggered intracellular free calcium levels and the inhibition of GPCR-stimulated inositol phosphate accumulation suggest that the mechanism underlying its spasmolytic effect may potentially involve inhibiting IP-mediated calcium release from intracellular stores [42].

## 4. Clinical Evidence

### 4.1. Fibromyalgia

In a cross-sectional study on 156 patients with fibromyalgia (144 women and 12 men), the prevalent use of NSAIDs and metamizole provided maximal pain relief, which underscored the necessity for further randomised control studies to evaluate drug efficacy [43]. Limitations encompass recall bias, uneven drug distribution, non-compliance, overlapping medications, and placebo-related uncertainties.

### 4.2. Arthralgia

Brito et al.’s review [44] focused on managing mild and moderate arthralgia caused by Chikungunya. Metamizole and paracetamol were frequently employed as monotherapy, with constant monitoring of adverse effects considered unnecessary in standard dosing. A case report on the management of a pregnant patient with type I osteogenesis imperfecta using quantitative ultrasonometry stated that at 31 weeks and 2 days of gestation, in addition to physiotherapy, only metamizole was successful in alleviating the patient’s pain, with the patient’s mobility being adequate [45].

### 4.3. Management of Acute and Chronic Pain

In a survey involving German anaesthesiologists and pain physicians, responses from 2237 participants revealed that 93.8% used metamizole for acute pain, and 76.7% used oral metamizole combined with other non-opioid analgesics for chronic pain [46]. Agranulocytosis occurred in 3.5% and 1.5% of respondents in acute and chronic pain management, respectively [46]. Limitations include the absence of specific patient numbers and information on severe side effects [46]. A cohort study on the use of tramadol and other analgesics as a risk factor for opioid use in 12,783 patients who were treated for pain stated that metamizole was the most frequent drug used and those who received tramadol were at risk of receiving opioids within 12 months [47]. The analgesic effects of metamizole and NSAIDs were similar. Limitations of the study included lack of access to medical records to verify indications to use different analgesics, the possibility of residual confounders, inability to assess whether the drug was brought from outside the health system, and use of psychoactive substances by the patient. A study comparing the tolerance of metamizole and morphine in 16 patients with acute pancreatitis showed faster onset of pain relief in those taking metamizole, with 75% of the patients attaining pain relief compared to 37.5% of those taking morphine [48]. In the end, 75% and 50% of the patients taking metamizole and morphine achieved pain relief, respectively [48].

### 4.4. Sciatic Pain

A multicentre observer-blind randomised trial [49] enrolled 260 patients with low back or sciatic pain. Metamizole administration resulted in significant pain reduction compared to diclofenac or placebo. Adverse reactions were most prevalent in the metamizole group (5%), compared to diclofenac (1%) and placebo (2%). An experimental study [32] on the effect of intraperitoneal administration of metamizole in relieving pain from chronic constriction injury of the sciatic nerve in Wistar rats showed a diminished development of neuropathic pain by reduced expression of pronociceptive interleukins.

### 4.5. Colicky Pain

A double-blind, double-dummy randomised controlled clinical trial [50] compared the analgesic effect of 1 or 2 g metamizole and 75 mg of sodium diclofenac administered intramuscularly or intravenously in 293 patients. Metamizole 2 g intramuscular demonstrated a superior analgesic effect from 60 min to 6 h than metamizole 1 g and sodium diclofenac 75 mg. The analgesic effect of metamizole 2 g intravenous was higher than intramuscular, with the effect starting at 10 and 20 min. Pain relief was proportionately higher in metamizole, with 2 g intravenous showing the most significant reduction in pain. A clinical pilot study [51] comparing the analgesic effects of cizolirtine citrate and metamizole sodium in 64 patients aged 18–65 with haematuria and moderate-to-severe pain showed a quicker onset of pain relief in those taking metamizole. A mean decrease of pain scores by 69.1% was observed in those taking metamizole compared with a 57.2% decrease in those taking cizolirtine citrate. At the 30-min mark, 56.3% stated complete, and 75% stated satisfactory pain relief, with fewer taking rescue medication when administered with metamizole. A limitation of the study is the lack of internal sensitivity measurement; hence, sensitivity to treatment differences was not ensured. A multicentre double-blind, randomised control parallel-group trial [52] comparing the efficacy of intravenous bolus of dexketoprofen trometamol and intravenous infusion of metamizole in 308 patients with moderate-to-severe pain caused by renal colic reported similar total pain relief scores (TOTPAR) scores to be identical in metamizole (15.5 ± 8.6) and dexketoprofen 50 mg (15.3 ± 8.6) and higher than dexketoprofen 25 mg (13.5 ± 8.6) with faster effects of analgesia occurring in those taking dexketoprofen with the efficacy between the two drugs being similar.

### 4.6. Postoperative Pain

In a prospective, placebo-controlled, randomised, double-blind trial [53] comparing the effects of intravenous 1 g metamizole, 1 g paracetamol, 8 mg lornoxicam, and 0.9% isotonic saline (placebo) on postoperative pain control and morphine consumption after lumbar disc surgery in 80 patients, pain reduction was observed in those administered metamizole and paracetamol. Still, it was less in those administered lornoxicam. ANOVA measures revealed that pain scores were significantly lower in metamizole than in lornoxicam. Limitations of the study included pain assessment only at rest, discharged 1 day after the procedure, and categorical evaluation of side effects not used to detect adverse effects caused by metamizole. In a double-blinded randomised control trial [54] assessing the analgesic effect of 500 mg metamizole and 500 mg aspirin in postoperative orthopaedic patients (281 patients), pain relief starting at 30 min after drug administration up to 6 h favoured metamizole over aspirin. Gastrointestinal side effects of metamizole were far less than those of aspirin.

In a prospective, randomised, double-blinded study of tramadol, metamizole, and paracetamol [55], assessing postoperative analgesia at home after ambulatory hand surgery in 120 patients, the number of patients requiring supplementary analgesics was 23% with tramadol, 31% with metamizole, and 42% with paracetamol. Those receiving metamizole and paracetamol had reasonable analgesia rates of 70% and 60%. Metamizole provided adequate analgesia for 69% of patients on day 1 and 85% on day 2. In a prospective, double-blinded, randomised study [56] on the management of postoperative pain after total hip arthroplasty, comparing the effects of metamizole and paracetamol on 110 patients, the mean values of pain AUC were 17.9 for metamizole and 30.6 for paracetamol during the first 24 h in the postoperative period with metamizole showing better pain control in the first 24 h. The limitation of the study is using a visual analogue scale (VAS) to measure pain, because various psychological and other factors influence it. A randomised, double-blind study [57] comparing the efficacy of intravenous paracetamol (1 g every 6 h) to metamizole (1 g every 6 h) and parecoxib (40 mg every 12 h) for postoperative pain relief after minor-to-intermediate surgery conducted on 196 patients, with patient-controlled piritramide as the rescue medication, showed a significant and quicker onset decrease in VAS pain scores on that administered metamizole. A lesser proportion of patients needed rescue medication, and the duration of the administration of metamizole and rescue medication was longer.

A double-blind, randomised controlled trial [58] reporting the mean difference in the pain score between metamizole, paracetamol, and ibuprofen found non-inferiority between the three drugs. However, the intake of rescue medication was higher in those taking an ibuprofen–paracetamol combination on POD 2. The study suggested metamizole as a valuable alternative to ibuprofen, especially in those with contraindications to NSAIDs. Limitations of the study included a lack of firm conclusions about medical safety, intake of tramadol at home by patients was significantly higher than the control group, rigorous exclusion criteria considerably reduced the sample size, and four different types of surgery were included that may all have various pain trajectories.

### 4.7. Pyrexia

In an open non-comparative study [59], 100 children (51 male, 49 female) aged 3 months–12 years, with an oral temperature of 38.5 degrees Celsius and complaints of pain from various causes, were given metamizole 10–15 mg/kg 6 to 8 hourly for 3 days. Of those with pain, 57% responded well and 43% responded satisfactorily to the drug. In those with fever, 66.7% showed a good response, 25.8% showed a satisfactory response, and 7.5% showed an unsatisfactory response.

### 4.8. Cancer Pain

In a double-blind, randomised parallel clinical trial [60] comparing the efficacy and tolerance of oral metamizole and morphine in 121 patients, the degree of pain relief using the 100 mm VAS showed comparable pain relief between those administered 2 g of metamizole and 10 mg of morphine. More patients had better tolerance and fewer side effects when taking metamizole. However, the onset of pain relief was faster in patients administered morphine.

### 4.9. Experimental Pain

**Figure 2:**
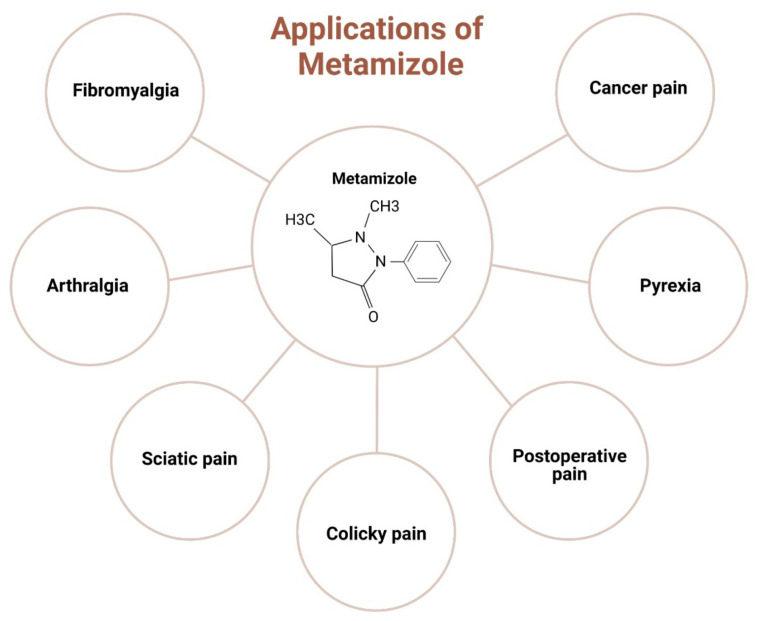
Clinical applications of metamizole in orthopaedics.

A randomised double-blind, parallel-group pre-test-post-test study [61] on the effect of tilidine and metamizole on cold pressor pain conducted in 264 healthy volunteers showed a lower AUPC% and higher pain tolerance compared with low-dose opioids, with fewer side effects than other drugs. A recent study [58] on 10 healthy volunteers (4 females and 6 males) comparing the analgesic efficacy of metamizole and tramadol in experimental pain showed the efficacy of tramadol to be much higher than metamizole, with the relative potencies of metamizole and tramadol assessed to be 1:23. However, no side effects were reported after the intake of metamizole. The clinical studies of metamizole are tabulated in Table 1. The clinical application of metamizole in orthopaedics is depicted in Figure 2.

## 5. Efficacy of Metamizole

With its role in various proposed pathways, metamizole affects antinociception, antipyretic analgesia, and antispasmolytic effects. The efficacy of the drug indicates a strong correlation between the dose of the drug and the onset of the action, duration, and degree of pain relief. Metamizole has also proved to be quite effective, with incidences of drug withdrawal being lower than other drugs of similar efficacy. In a comparative study [55] exploring the analgesic efficacy of tramadol, metamizole, and paracetamol, 81% of study subjects on day 1 and 82% on day 2 deemed the drug effective in providing pain relief following ambulatory hand surgery, with a satisfaction rate of 59% in individuals administered metamizole. Dose-dependent analgesic effects were identified, indicating that subjects receiving 15 mg of metamizole consumed 3.85 mg of morphine.

In comparison, those administered 40 mg of metamizole consumed only 2.55 mg of morphine, thus potentially minimising the adverse effects associated with morphine [62]. In adult acute renal colic, where analgesics such as cizolirtine citrate or metamizole sodium were employed, a swift onset of pain relief was particularly evident with metamizole. Notably, a mean decrease in pain scores by 69.1% was observed, and at the 30-min mark, 56.3% of patients reported complete pain relief, with 75% expressing satisfactory pain relief. Additionally, fewer individuals required rescue medication when metamizole was administered [51]. The role of metamizole in postoperative pain management following total hip arthroplasty showcased its potent efficacy, with lower pain scores recorded on the VAS, indicating a mean pain value of 17.9. Patients receiving metamizole also required a lower dose of morphine [56].

In treating lower third molar surgery, administering 2 g metamizole significantly reduces VAS pain scores starting from 15 min. At the 60-min mark, a notable percentage of patients achieved a 50% reduction in their basal VAS scores. The therapeutic effect was reported as “excellent” in 50.7% of patients and 41.7% of physicians, with a higher percentage of patients experiencing at least a one-grade improvement in verbal ratings compared to baseline values [63]. Metamizole’s effectiveness in managing pain post minor-to-intermediate surgeries was also evident, indicating a substantial reduction in pain scores [56]. Cancer patients receiving oral metamizole at a dosage of 2 g demonstrated a marked decrease in baseline VAS pain scores from 81.8 ± 0.6 to 34.9 ± 25.8 within 7 days, with the most significant pain improvement observed in the metamizole 2 g group by the fifth day [60]. A reduction focusing on oral metamizole monotherapy revealed that the number needed to treat to achieve a 50% reduction in pain after minor surgeries was 2.1 for metamizole 500 mg, showcasing its superior efficacy compared to other drugs [66].

In individuals treated with 800 mg of metamizole for cold pressor pain, a significantly lower area percentage under pain curve (%AUPC) ratings and higher pain tolerance were observed compared to low-dose opioid and control groups [61]. An ANOVA comparison between metamizole, paracetamol, and lornoxicam indicated significantly lower pain scores in those treated with metamizole post-lumbar disc surgery [53]. From the above studies, it is inferred that metamizole effectively manages pain and pyrexia. In most cases, patients were provided with satisfactory and good pain relief with the early start of its analgesic effects. It provided the patients with almost seamless postoperative analgesia in a few cases. Metamizole also reduces the unpleasant side effects of narcotic drugs and the need for rescue medications that some patients have to take to cope with the pain, one of the critical features highlighting the drug in a positive light. Early relief from pain also helps the patient return to their earlier lifestyle effortlessly. The efficacy of metamizole in the published literature is tabulated in Table 2.

## 6. Safety Profile of Metamizole

Despite providing effective pain relief from musculoskeletal disorders, metamizole faced withdrawal from the markets in several countries, including the USA, UK, Canada, Australia, Japan, Sweden, Denmark, and India, following ongoing safety debates [57]. Notably, when administered in appropriate doses, it exhibits fewer gastrointestinal and renal side effects; however, instances of agranulocytosis, neutropenia, and various blood disorders have been reported [57]. Categorised as a Category B drug capable of inducing drug-induced liver injury, metamizole raised concerns with 40 reported incidents globally [67,68]. Cases of metamizole-induced liver injury revealed findings such as low-grade fibrosis (29%) and extensive centrilobular necrosis (35%) [68]. A database search identified 143 reports on liver-related side effects associated with metamizole, with the three leading causes being hepatic failure, drug-induced liver injury, and jaundice [68].

Among the most severe complications tied to metamizole are agranulocytosis and anaphylactic shock. Combined use with drugs such as methotrexate increases the risk of agranulocytosis [19,69]. First detected in 1935, agranulocytosis linked to dipyrone raises concerns, given its structural similarity to aminopyrine, a previously discontinued analgesia associated with increased agranulocytosis rates [70]. Reports indicate agranulocytosis after administering a test dose [71], highlighting immediate cell destruction in internalised individuals [72]. Factors related to agranulocytosis include the history of using a new medication or change in the previous medication, exposure to chemical or physical agents recently, or a recent bacterial or viral infection [71]. Prognosis is often decreased by age over 65 years, neutrophil count at diagnosis less than <0.1 × 10^9^/L, presenting with severe deep infections or septicaemia or septic shock, and underlying disease or severe comorbidity. However, with appropriate management, mortality from idiosyncratic drug reactions stands at approximately 5% [73]. Agranulocytosis can also be successfully treated with recombinant human granulocyte colony-stimulating factors [74].

Extended use of metamizole may induce renal toxicity, with allergy-related manifestations such as skin rashes and asthma. Risk factors include intolerance to metamizole, other non-opioids, and existing bronchial asthma. Notably, the incidence of agranulocytosis is minimal in patients concurrently receiving antibiotics [69]. In Europe, the annual incidence of drug-induced agranulocytosis ranges from 3.4 to 5.3 cases per million population, while in the USA, it is between 2.4 and 15.4 cases per million per year [69]. The drug’s impact on bone marrow, leading to blood dyscrasias such as leukopenia and aplastic anaemia, involves genetic mechanisms, with incidence variations in different geographical regions [69,75]. Complications such as angioedema and urticaria induced by pyrazolone derivatives may stem from a pseudo-allergic reaction, likely occurring via COX inhibition. Severe outcomes include Stevens–Johnson syndrome and toxic epidermal necrolysis [69,76]. While rare, reports of acute kidney injury associated with metamizole exist, with a generally reduced prognosis compared to NSAIDs’ impact on the kidneys. The drug’s role in obstructive reactions leading to asthma attacks in allergic individuals involves the production of cysteinyl leukotrienes [69].

Metamizole, despite its potential toxicity, appears less harmful compared to NSAIDs. Unlike NSAIDs, which exhibit various side effects involving the gastrointestinal, renal, and cardiovascular systems, metamizole, with its mechanism redirecting prostaglandin synthesis, demonstrates a protective effect on the gastric mucosa [77,78]. NSAIDs’ inhibition of COX 1-mediated generation of cytoprotective prostanoids like PGE2 and PGI2 contributes to their adverse effects [79]. Previous evidence highlighted significant renal risks and arrhythmias associated with NSAIDs, particularly rofecoxib, with effects intensifying with a higher dosage and a longer duration [77]. Another meta-analysis reported increased hypertension incidence with COXIBs compared to non-selective NSAIDs [80]. Studies on celecoxib underscore the elevated risk of cardiovascular complications, indicating a dose-related response to toxicity [81,82]. Paracetamol, while commonly used, has raised concerns regarding its impact on neurodevelopment. Observational studies have demonstrated a correlation between paracetamol use and neurodevelopmental disorders when taken for an extended duration during pregnancy. Issues related to gross motor development, communication, externalising and internalising behaviours, higher activity levels, hyperkinetic disorder, and the occurrence of attention deficit hyperactivity disorder (ADHD) by age seven have been identified [36,83]. These findings highlight the need for careful consideration when prescribing paracetamol during pregnancy and suggest a potential link between its prolonged use and adverse neurodevelopmental outcomes in children.

In contrast to NSAIDs, metamizole’s complications, primarily involving agranulocytosis, various allergic reactions, and liver toxicity, position it as a comparatively less toxic option [69]. Observational and cross-sectional studies suggest a correlation between paracetamol use and asthma diagnosis or exacerbations, raising concerns about its safety, particularly during pregnancy [84,85,86]. When considering and comparing the reporting odds ratio (ROR) between metamizole and NSAIDs in gastric or duodenal ulcers, metamizole showed an ROR of [95% CI]: 0.9 [0.7–1.2] when compared to non-selective NSAIDs (ROR [95% CI] ibuprofen 8.3 [7.8–8.7], naproxen 10.7 [10.2–11.1], and diclofenac 14.3 [13.8–14.9]) and in selective NSAIDs (celecoxib 6.9 [6.5–7.3], etoricoxib 7.2 [6.4–8.2]). However, there is a slightly increased risk for upper gastrointestinal bleeding in metamizole (ROR [95% CI] 1.5 [1.3–1.7]), which is still lower when compared to non-selective NSAIDs (ROR [95% CI] ibuprofen 8.2 [8.0–8.5], naproxen 7.9 [7.7–8.1], and diclofenac 9.1 [8.8–9.3]) and non-selective NSAIDs (ROR celecoxib 5.9 [5.7–6.1] and etoricoxib 5.8 [5.2–6.4]). When comparing the significance of renal impairment, it was also lesser in those taking metamizole (ROR [CI 95%] 1.2 [1.0–1.3]) when compared to NSAIDS (ROR [CI 95%] ibuprofen 2.4 [2.3–2.5], diclofenac 2.3 [2.2–2.4], celecoxib 2.1 [2.0–2.2], etoricoxib 1.9 [1.7–2.2]). When comparing the cardiovascular effects between the two, metamizole showed an ROR of [CI 95%] 0.5 [0.4–0.5] when compared to selective COX 2 inhibitors (ROR [CI 95%] for celecoxib 8.5 [8.3–8.7] and etoricoxib 1.9 [1.7–2.2]) [24]. When comparing metamizole with paracetamol, the risk ratio (95% CI) between the two for adverse effects was 1.08 (0.69,1.68) and dropped out due to adverse effects was 1.47 (0.32,6.75), pain at the site of injection was 0.50 (0.14–0.79), nausea was 1.17 (0.74–1.6), vomiting was 0.74 (0.34–1.62), exclusively blood dyscrasias was 2 (0.20–20.33), cardiovascular effects was 3.48 (1.07–11.27), neurological effects was 0.63 (0.08,4.94) and dermatologic–l conditions was1.44 (0.24,8.55) [87]. The side effects overall are lesser in those taking metamizole.

## 7. Comparative Investigations

A study systematically compared the analgesic effect of tramadol, metamizole, and paracetamol which indicated that 81% of study subjects on day 1 and 82% on day 2, compared to 52% on day 1 and 2 in those taking tramadol, perceived metamizole as providing adequate pain relief following ambulatory hand surgery. Notably, 59% of patients expressed satisfaction with metamizole compared to only 47% of those taking tramadol [55]. In adult acute renal colic cases, the onset of pain relief was notably quicker with metamizole. There was a 69.1% and 57.2% decrease in the VAS pain score intensity in those taking metamizole and cizolirtine citrate, respectively. At the 30-min mark, in those taking metamizole and cizolirtine citrate, respectively, 56.3% and 48.4% expressed complete pain relief, and 75% and 64.5% expressed satisfactory pain relief [51]. Patients undergoing postoperative pain management after total hip arthroplasty demonstrated reduced outcomes, with reduced morphine consumption and lower pain scores of 17.9 in those taking metamizole when compared to a score of 30.6 in those taking paracetamol. This suggests the drug’s potential as an effective analgesic in orthopaedic surgery [56]. The use of 2 g of metamizole in lower third molar surgery revealed significant reductions in VAS pain scores starting as early as 15 min after administration, with a decrease in pain by 37.3% in those taking 2 g of metamizole when compared to a 20.9% decrease in those taking ibuprofen. This was further supported by a higher percentage of patients experiencing a meaningful reduction in pain, underlining the drug’s rapid onset and efficacy in this specific surgical procedure [3]. After minor-to-intermediate surgery, metamizole exhibited a significant decrease in pain scores compared to other analgesic groups. Scores after 1 week were 7.1 and 12.2, and the values of associated pain were documented to be 5.6 and 12.4 in metamizole and parecoxib, respectively [56].

Treatment of cancer pain indicated a decrease in baseline VAS pain scores from 81.8 ± 0.6 to 34.9 ± 25.8 in 7 days, with the highest pain improvement in the metamizole 2 g group by the fifth day. However, a slight difference was found in those taking metamizole 2 g and morphine. However, this difference was not statistically significant [60]. Patients treated with 800 mg of metamizole for cold pressor pain experienced slightly lower area percentage under the pain curve (%AUPC) ratings and a higher pain tolerance compared to low-dose opioid and control groups (59.7 ± 26.0, 71.1 ± 22.4, 62.1 ± 24.7), further substantiating its efficacy in experimental pain settings [61]. ANOVA scores comparing metamizole, paracetamol, and lornoxicam revealed significantly lower pain scores in individuals administered metamizole post-lumbar disc surgery [53]. However, other experiments have shown that lornoxicam is non-inferior compared to other drugs. The need for rescue medication was elevated in those taking lornoxicam [88].

Metamizole’s efficacy is evident in reducing pain scores across multiple studies, highlighting its role in diverse clinical contexts. Findings from various studies indicate increased incidences of gastrointestinal side effects in patients taking NSAIDs, morphine, and tramadol, and increased bilirubin values were found in those taking cizolirtine citrate. Tiredness and sleepiness were found in patients taking metamizole. Syncope occurred in those taking ibuprofen. While some studies emphasise the economic advantage of metamizole over other analgesics, concerns about safety, including agranulocytosis risk, require careful consideration—the multifaceted evidence positions metamizole as a valuable analgesic option, especially in specific clinical scenarios. The comparative analysis of metamizole with other drugs is tabulated in Table 3.

## 8. Challenges and Future Directions

**Figure 3:**
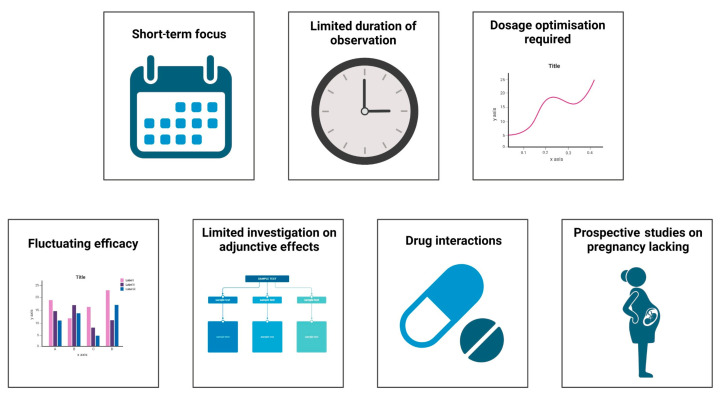
Challenges in current research of metamizole.

The critical challenges in the current research on metamizole in various clinical conditions are depicted in Figure 3. Currently, most articles on metamizole are randomised control studies, comparative studies, and systematic reviews with a few cross-sectional studies. These studies only identify the short-term effectiveness of the drug in pain management and highlight the acute onset of side effects such as agranulocytosis. Few long-term cohort and prospective studies highlight the drug’s long-term effectiveness and impact on various organs. Most studies only follow the patient for a week at a maximum, which is inadequate for major surgeries. In addition to this, patients were often given some dosage of the drug despite undergoing different surgical procedures. Certain studies assessed pain only at rest, and the patient’s mobility after treatment was not reported. Studies have also unequally distributed the drug amongst participants, which may add as a source of error in calculating the efficacy of the number of side effects produced by the drug.

Despite various comparative studies between the drugs, the drug’s efficacy kept fluctuating. Adverse effects of combinations of drugs have not been extensively studied. Francisco Javier Lopez-Munoz et al. [89] performed a study on improving antinociception while decreasing the impact of constipation by combining metamizole and tramadol in arthritic rats. Furthermore, there have been limited investigations on drug interactions and their impact on efficacy. Prospective studies on the effects of the drug on pregnant mothers, such as those performed by Katarina Dathe et al., are required, because there have been varying impacts on the impact of the drug on the fetus whilst taking the drug during pregnancy [90,91].

## 9. Conclusions

Metamizole has been critical in managing pain in musculoskeletal disorders, pyrexia, and analgesia for over 90 years. Despite its usage since 1920, the drug’s mechanism of action continues to be elusive, with many theories being put forward, cementing its role in antinociception, analgesic, and antispasmolytic effects. Despite various articles suggesting the effective use of the drug in pain management, fluctuating results between different studies raise questions about the efficacy of the drug. However, the drug has been consistent in reducing the number of narcotic analgesics consumed and increasing the duration between the administration of analgesics and the demand for narcotic analgesics, thus effectively reducing the incidence of side effects from consumption of narcotic analgesics. Furthermore, the drug’s ability to redirect prostaglandin synthesis protects against gastric side effects. Despite having fewer adverse effects when compared to other drugs of similar efficacy, reports on agranulocytosis have become a prime reason for the withdrawal of the drug from various markets. Although many countries are wary of the drug’s efficacy and side effect profile, this review explores a more comprehensive aspect focused on multiple dimensions of drug application and findings found thus far regarding its usage, efficacy, and side effect profile. More conclusive research on the side effects of the drug would shed light on ways to decrease the incidence of the side effects and further increase the drug’s efficacy.

## Figures and Tables

**Table 1:** Clinical studies of metamizole.

Study (Ref.)	Study Type	Design	Study Population	Outcome	Limitation
Analgesic medication in fibromyalgia patients: a cross-sectional study [43]	Cross-sectional study	Patients were questioned on their current medication, dose, effect, indication, and treatment regimen	Patients were recruited from the Department of Neurology, University Hospital Würzburg, Germany, between 2014 and 2019 156 patients; 144 women and 12 men were included	A higher frequency of patients used NSAIDs and metamizole with a greater proportion of people discontinuing NSAIDs due to a lack of effect of the drug	Recall bias, Uneven drug distribution, Non-compliance, Overlapping medications, Placebo-related uncertainties
Pharmacologic management of pain in patients with Chikungunya: a guideline [44]	Review article	-	-	Were stated to be good analgesics when administered in appropriate doses at regular intervals	-
Clinical management of a pregnant patient with type I osteogenesis imperfecta using quantitative ultrasonometry—a case report [45]	Case report	-	As a result of pre-term labour and pregnancy-related hyperlordosis, the patient was immobilised for 3 weeks at 21 weeks of gestation, which caused an increase in pain of the lumbar spine and arthralgia of the hip joints and hospitalization at 31 weeks and 2 days of gestation	In addition to physiotherapy, metamizole helped alleviate the pain	-
Dipyrone is the preferred nonopioid analgesic for the treatment of acute and chronic pain. A survey of clinical practice in German-speaking countries [46]	Questionnaire-based study	A link to a questionnaire was sent to anaesthesiologists and pain physicians via mail	A total of 2237 responses were analysed, 94.7% used non-opioid analgesics in which 93.8% administered metamizole, 54% administered NSAIDs, 41.8% administered COX-2 inhibitors and, 49.2% administered paracetamol	Metamizole was concluded to be the preferred analgesic in peri-operative and chronic pain settings	Absence of specific patient numbers, Lack of information on severe side effects
Use of tramadol or other analgesics in patients treated in the emergency department as a risk factor for opioid use [47]	Cohort study	3 groups Mean dosage +/− SD (mg) Tramadol (60.0 +/− 29.7) NSAIDs Diclofenac Sodium (75.9 +/− 10.1) Acetaminophen (2689+/− 2339) Metamizole (1599 +/− 1100)	Patients arriving at the emergency department Total 12,783 Tramadol n = 1454 NSAIDs n = 5309 Dipyrone n = 6020	Efficacy of metamizole and NSAIDs similar Those taking tramadol at risk of receiving morphine >/= 10 mg in following 12 months	Lack of access to medical records to verify indications for analgesics, Possibility of residual confounders, Possibility of drug being bought outside healthcare, Use of other psychoactive substances
Efficacy and tolerance of metamizole versus morphine for acute pancreatitis pain [48]	Pilot, randomised, active controlled, open clinical trial	2 groups Morphine (Oglos) 1% 10 mg/kg 4 h s.c Metamizole (Nolotil) 2 g/8 h i.v slow perfusion for 3 min	Patients between the ages of 18 and 75 of both sexes with acute pancreatitis getting admitted within 12 h of onset of symptoms	Efficacy of metamizole was more than morphine with patients attaining greater pain relief	-
Parenteral dipyrone versus diclofenac and placebo in patients with acute lumbago or sciatic pain: randomized observer-blind multicentre study [49]	Multicentre, observer-blind randomised trial	Administered drugs for 1–2 days Metamizole 2.5 g Diclofenac 75 mg Placebo	260 patients enrolled into the study Divided into 3 groups Metamizole Diclofenac Placebo	Significant pain reduction in those taking metamizole. % of patients stating drug to be satisfactory of very good Metamizole (59%) Diclofenac (30%) Placebo (18%)	None mentioned
Metamizole relieves pain by influencing cytokine levels in dorsal root ganglia in a rat model of neuropathic pain [32]	Experimental study	Chronic constriction injury of sciatic nerve deliberately induced metamizole (Met, 500 g/kg; SANOFI, Germany) dissolved in water and administered intraperitoneally pre-emptively 16 and 1 h before CCI and then twice for 7 days	Male Wistar rats (270–300 g) Divided into 2 groups 1 group receiving metamizole and the other group water	Diminished development of neuropathic pain symptoms with inhibiting expression of pronociceptive interleukins and chemokines	None mentioned
Comparison of the onset and duration of the analgesic effect of dipyrone, 1 or 2 g, by the intramuscular or intravenous route, in acute renal colic [50]	Double-dummy randomised controlled clinical trial	Patients between the ages of 18–70 years diagnosed with renal colic between December 1988 and March 1991 Divided into 6 groups and administered drugs via i.m or i.v route Group 1—metamizole 1 g i.m Group 2—metamizole 1 g i.v Group 3—metamizole 2 g i.m Group 4—metamizole 2 g i.v Group 5—diclofenac sodium 75 mg i.m Group 6—diclofenac sodium 75 mg i.v	Total 293 patients Group 1 n = 71 Group 2 n = 30 Group 3 n = 67 Group 4 n = 71 Group 5 n = 32 Group 6 n= 22	Analgesic effects in those taking metamizole 2 g i.v was highest when compared to other groups	-
Comparison of cizolirtine citrate and metamizole sodium in the treatment of adult acute renal colic: A randomized, double-blind, clinical pilot study [51]	A randomised, double-blind, clinical pilot study	conducted in the emergency department of 6 Czech Republic hospitals between October 2000 and February 2001 Patients of both sexes between the ages of 18–65 were divided into 2 groups and administered single i.v dose of either 350 mg of cizolirtine citrate or 2500 mg of metamizole	Total n = 63 Cizolirtine citrate n = 31 Metamizole n = 32	Proportion of patients stating satisfactory and complete pain relief at the 30-min mark was higher in those taking metamizole	Lack of internal sensitivity measurement, sensitivity to treatment differences is not ensured
Comparison of intravenous dexketoprofen and dipyrone in acute renal colic [52]	Multicentre double-blind randomised control parallel group trial	Patients were recruited from 18 centres in Spain, Finland, and Sweden from June 1998 to September 1999 of both sexes between the ages 18 and 70 years were divided into 3 groups and were administered dexketoprofen trometamol 25 and 50 mg as solutions of 1 and 2 mL and metamizole 2 g in the form of 5 mL ampules	Total n = 333 Dexketoprofen Trometamol 25 mg n = 112 Dexketoprofen Trometamol 50 mg n = 113 Metamizole n = 108	Efficacy of 2 drugs were equal with faster effects of analgesia occurring in those taking dexketoprofen trometamol	-
Efficacy of intravenous paracetamol, metamizole and lornoxicam on postoperative pain and morphine consumption after lumbar disc surgery [53]	Prospective, placebo-controlled randomised double-blind study	80 patients scheduled for elective lumbar disc surgery under general anaesthesia were divided into 4 groups and were administered supplemental i.v injections of 1 mg metamizole or 1 mg paracetamol or 8 mg lornoxicam or 0.9% isotonic saline	Total n = 80 Metamizole n = 18 Paracetamol n = 20 Lornoxicam n = 20 Control. n = 19	ANOVA scores revealed pain scores of metamizole to be much lower than lornoxicam	Pain was assessed only at rest, Patients were discharged 1 day after the surgery, Categorical evaluation of side effects caused by metamizole had not used
Analgesic effectiveness of dipyrone (metamizole) for postoperative pain after herniorrhaphy: a randomized, double-blind, dose-response study [62]	Double-blind, randomised trial	162 patients undergoing elective inguinal, umbilical or epigastric herniorrhaphy were divided into 2 groups and were administered metamizole of either 15 mg/kg or 40 mg/kg dose	Total n = 162 D15 n = 82 D40 n = 80	Higher dose led to movement induced constipation and fewer opioid related side effects	Short period of observation
A randomized double-blind placebo-controlled study of dipyrone and aspirin in post-operative orthopaedic patients [54]	Randomised double-blind placebo-controlled study	Patients with sustained long bone fracture underwent surgical manipulation and closed reduction of fracture under general anaesthesia were divided into 3 groups and received either 500 mg metamizole, 500 mg aspirin or placebo	Total n = 254 Metamizole n = 91 Male n = 74 Female n = 17; Aspirin n = 93 Male n = 65 Female n = 26 2 patients lacked sex record; Placebo = 70 Male n = 54 Female n = 16	Pain relief was faster in onset and greater in those taking metamizole	-
Postoperative analgesia at home after ambulatory hand surgery: a controlled comparison of tramadol, metamizole, and paracetamol [55]	Prospective, randomised, double-blinded study	120 patients who had undergone hand surgery were allocated in equal sizes to 3 groups receiving Paracetamol 1 g every 6 h Metamizole 1 g every 6 h Tramadol 100 mg every 6 h	Total n =120 Paracetamol n = 40 Metamizole n = 40 Tramadol n = 40	Adequate efficacy was seen on those taking metamizole	-
Treatment of postoperative pain after total hip arthroplasty: comparison between metamizole and paracetamol as adjunctive to opioid analgesics—prospective, double-blind, randomised study [56]	Prospective, double-blind randomised study	110 patients were divided into 2 groups receiving either metamizole i.v 1.5 g every 8 h or paracetamol 1 g every 8 h during the first 24 h	Total n = 94 Metamizole n = 51 Paracetamol n = 43	Mean values of pain AUC were lower for metamizole	Measuring pain using the VAS scale due to it be influenced by various psychological and other factors
Efficacy of intravenous paracetamol compared to dipyrone and parecoxib for postoperative pain management after minor-to-intermediate surgery: a randomised, double-blind trial [57]	Randomised, double-blind trial	222 patients were recruited and were assigned to 4 groups and received either i.v paracetamol 1 g every 6 h, metamizole 1 g every 6 h, parecoxib 40 mg every 12 h or placebo (0.9% saline)	Total n = 196 Paracetamol n = 49 Metamizole n = 49 Parecoxib n = 49 Placebo n = 49	Lesser proportion of patients asked and demanded for rescue medication with a longer duration between the administration of metamizole and the rescue medication	-
Metamizole vs. ibuprofen at home after day case surgery: A double-blind randomised controlled noninferiority trial [58]	Investigator-initiated, double-blind, randomised controlled, non-inferiority trial	200 patients undergoing elective arthroscopic shoulder, haemorrhoid, or knee surgery or inguinal hernia repair were randomised to receive either a combination of metamizole and paracetamol 1 g orally, thrice daily or ibuprofen and paracetamol 600 mg orally thrice daily for 4 days	Total n = 200 Metamizole and paracetamol n = 100 Ibuprofen and paracetamol n = 100	Similar efficacy of both drugs. On postoperative day 2, patients taking ibuprofen required rescue medication. Metamizole acts as a valuable replacement for NSAIDs in the event of a contraindication	Lack of firm conclusions about medical safety, Intake of tramadol by patients at home was significantly higher than control group, Rigorous exclusion criteria leading to a decrease in sample size
Metamizole in pain and fever [59]	Open non-comparative study	100 children in Ganga Ram Hospital, Lahore, aged between 3 months to 12 years with an oral temperature of 38.5 degree were included	Children were administered metamizole 10–15 mg/kg 6 to 8 hourly for 3 days	For those in pain, 57% showed a good response and 43% showed a satisfactory response. For those with fever, 66.7% showed a good response and 25.8% showed a satisfactory response.	-
Oral metamizole (1 g and 2 g) versus ibuprofen and placebo in the treatment of lower third molar surgery pain: randomised double-blind multi-centre study. Cooperative Study Group [63]	Double-blind, double-dummy, randomised, controlled clinical trial	Between January 1994 and January 1995, patients between the ages of 18 and 60 of both sexes who had undergone extraction of the lower third molar were assigned to 4 groups and were provided with either a single oral dose of 1 g or 2 g metamizole in a new galenic form, Ibuprofen 600 mg or placebo	Total n = 253 Metamizole 1 g n = 75 Metamizole 2 g n = 72 Ibuprofen 600 mg n = 74 Placebo n = 32	Quicker and more effective analgesia was achieved with those taking metamizole 2 g. No significant differences were found between those taking metamizole 1 g and 2 g.	-
Is dipyrone effective as a pre-emptive analgesic in third molar surgery? A pilot study [64]	Pilot prospective double-blind placebo-controlled study	36 patients in need of oral and maxillofacial surgery were selected and were divided into study and control groups	Study group was administered metamizole preoperatively and the control group was administered metamizole in the immediate postoperative period	Improved efficacy was seen when the drug was administered pre-emptively	-
Efficacy and tolerance of oral dipyrone versus oral morphine for cancer pain [60]	Double-blind, randomised, and parallel clinical trial	Between January 1991 and May 1992, patients of both sexes above the age of 18 years were included in a 7-day trial. Patients were assigned to 3 groups and were administered either metamizole 1 g (Nolotil, half an ampule) every 8 h, morphine 10 mg orally every 4 h or metamizole 2 g (Nolotil, an ampule) every 8 h	Total n = 121 Group 1 n = 41 Group 2 n = 42 Group 3 n = 38	Comparable pain relief in those taking metamizole 2 g and morphine 10 mg with an increased number of side effects seen in those taking morphine. Faster onset of pain relief seen in those taking morphine	-
Tilidine and dipyrone (metamizole) in cold pressor pain: A pooled analysis of efficacy, tolerability, and safety in healthy volunteers [61]	Randomised, double-blind, parallel group. Pre-test-post-test design.	264 healthy volunteers from 3 separate substudies Substudies 1 and 2 tilidine/naloxone combination was given (Tilidin comp. STADA 50 mg/4 mg per 0.72 mL, STADA Arzneimittel AG) Substudy 3 was given metamizole solution (Novaminsulfon-ratiopharm 500 mg/mL, Ratiopharm)	Substudies 1 and 2 Low dose opioid group n = 60 High dose opioid group n = 59 Pooled control group n = 58 Substudy 3 Metamizole group n = 40 Control group n = 41	%AUPC was in the following order from ascending to descending Low-dose opioid group Pooled control group of substudies 1 and 2 Control group of substudy 3 Metamizole group High-dose opioid group	-
Comparison of the analgesic efficacy of metamizole and tramadol in experimental pain [65]	Randomised double-blind study	Constant painful stimuli were applied by controlled electric stimulation of tooth pulp. Analgesia was monitored by verbal pain rating	10 healthy volunteers	Higher pain relief was attained in those taking tramadol. However, no side effects were reported with the intake of metamizole	-

**Table 2:** Efficacy of metamizole in published literature.

Study (Ref.)	Drug Evaluation Criteria: Conditions under Assessment	Efficacy
Analgesic medication in fibromyalgia patients: A cross-sectional study [43]	Assessing the efficacy of metamizole in managing pain in those with fibromyalgia syndrome	In accordance to the NRS scale pain, reduction in those taking metamizole was 2.0 (0–8)
Clinical management of a pregnant patient with type I osteogenesis imperfecta using quantitative ultrasonometry—a case report [45]	Managing a pregnant patient with osteogenesis type 1	Values had not been mentioned; however, in addition to physiotherapy, metamizole was the only drug that helped alleviate pain
Use of tramadol or other analgesics in patients treated in the emergency department as a risk factor for opioid use [47]	Assessing the risk of opioid usage within 12 months of initial administration of metamizole	Those taking metamizole received analgesics only on day 1 when compared to other groups who had receive additional doses in the following day
Efficacy and tolerance of metamizole versus morphine for acute pancreatitis pain [48]	Assessing the efficacy of metamizole in the management of acute pancreatitis pain	75% of patients taking metamizole achieved pain relief within the first 24 h. The mean time to achieve pain relief was 10 +/− 6.6. 75% of patients taking metamizole achieved pain relief
Parenteral dipyrone versus diclofenac and placebo in patients with acute lumbago or sciatic pain: randomized observer-blind multicentre study [49]	Assessing the analgesic effect of metamizole in lumbago or sciatic pain	A greater number of patients taking metamizole achieved significant pain relief at the end of 1 and 6 h. 59% of those taking metamizole stated the efficacy of the drug to be “very good” or “excellent”
Comparison of the onset and duration of the analgesic effect of dipyrone, 1 or 2 g, by the intramuscular or intravenous route, in acute renal colic [50]	Assessing the onset and duration of analgesia by metamizole in treating cancer pain	The pain scores at the 1 and 6 h mark were greatest in those taking dipyrone 2 g i.m
Comparison of cizolirtine citrate and metamizole sodium in the treatment of adult acute renal colic: A randomized, double-blind, clinical pilot study [51]	Assessing efficacy of the drug in treating adult acute renal colic	From a baseline VAS pain score of 82.59 at the 30-min mark, values were reduced to 25.41, which marks a decrease in pain scores by 69.1%
Comparison of intravenous dexketoprofen and dipyrone in acute renal colic [52]	Assessing the efficacy of metamizole in treating renal colic	Those taking metamizole showed a total sum of pain relief of 14.2+/− and pain intensity difference on the VAS scale as 58.6 +/− 22.7. 87% of patients attained pain relief scores below 50%
Efficacy of intravenous paracetamol, metamizole and lornoxicam on postoperative pain and morphine consumption after lumbar disc surgery [53]	Assessing postoperative pain and the degree of morphine consumption after lumbar disc surgery	During the first 24 h of the study period, pain was reduced in patients taking metamizole (*p* = 0.001) and paracetamol (*p* = 0.20) but not in those taking lornoxicam (*p* = 0.20) Pain scores in the metamizole group were significantly lower in metamizole when compared to lornoxicam (*p* = 0.031)
Analgesic effectiveness of dipyrone (metamizole) for postoperative pain after herniorrhaphy: a randomized, double-blind, dose-response study [62]	Assessing the analgesic efficacy of metamizole in treating postoperative pain after herniorrhaphy	The study showed a dose dependent efficacy with a lower incidence of moderate and severe pain in those taking 40 mg of metamizole and a lower cumulative effect of morphine consumption post-surgery
A randomized double-blind placebo-controlled study of dipyrone and aspirin in post-operative orthopaedic patients [54]	Assessing the analgesic effect of metamizole in postoperative orthopaedic patients	Onset of action was quick and higher values of relief from pain were achieved
Postoperative analgesia at home after ambulatory hand surgery: a controlled comparison of tramadol, metamizole, and paracetamol [55]	Assessing the efficacy of the drug in relieving postoperative analgesia after an ambulatory hand surgery	81% of patients taking metamizole on day 1 and 82% of patients taking the drug on day 2 stated adequate pain relief, with 59 of patients stating drug satisfaction
Treatment of postoperative pain after total hip arthroplasty: comparison between metamizole and paracetamol as adjunctive to opioid analgesics—prospective, double-blind, randomised study [56]	Assessing postoperative pain after hip arthroplasty	VAS pain values showed a statistically significant difference, which favoured metamizole at the 6 h mark (*p* = 0.038), 8 h mark (*p* = 0.036), 14 h mark (*p* = 0.011), and 22 h mark (*p* = 0.025) Mean cumulative pain values for metamizole and paracetamol were 17.9 and 30.6, respectively
Efficacy of intravenous paracetamol compared to dipyrone and parecoxib for postoperative pain management after minor-to-intermediate surgery: a randomised, double-blind trial [56]	Assessing the efficacy of the drug in the management of pain after minor-to-intermediate surgeries	Patients with pain scores more than 40 on VAS scale were taken in the study. Surgical pain postoperatively in the 1 h was highest in those taking placebo (33.8{19.2}) and lowest in those taking metamizole (25,7{15.3)}. Associated pain was once again highest in those taking placebo (25.4{16.6}) and lowest in those taking metamizole (9.0{11.7})
Metamizole in pain and fever [59]	Assessing the efficacy, safety, and tolerability in the management of pain and fever in children	66.7% patients showed a good response, 25.8% showed a satisfactory response, and 7.5% showed an unsatisfactory response
Oral metamizole (1 g and 2 g) versus ibuprofen and placebo in the treatment of lower third molar surgery pain: randomised double-blind multi-centre study. Cooperative Study Group [63]	Assessing the efficacy of metamizole in the treatment of surgery pain of the lower third molar	36.1% of those taking metamizole 2 g had a decrease of 50% of basal VAS scores within the first 15 min and 88.7% of patients within the 1 h mark. A sum pain intensity difference of 38.1(17.0) was achieved by those taking the drug
Is dipyrone effective as a pre-emptive analgesic in third molar surgery? A pilot study [64]	Assessing the effectiveness of metamizole as a pre-emptive analgesic in third molar surgery	The efficacy of metamizole was better in those taking the drug pre-emptively rather than in the immediate postoperative period
Efficacy and tolerance of oral dipyrone versus oral morphine for cancer pain [60]	Assessing the efficacy and tolerance of cancer pain	The mean percentage decrease in pain was highest in those taking 1 g of metamizole (64.4 +/− 37.9%) and lowest in those taking 10 mg of morphine (49.6 +/− 38.8%). There were no significant differences in pain intensity between the groups and percentage of pain improvement was significantly higher in those taking 10 mg of morphine and 2 g of metamizole at days 3 and 5
Tilidine and dipyrone (metamizole) in cold pressor pain: A pooled analysis of efficacy, tolerability, and safety in healthy volunteers [61]	Assessing the effect of metamizole in cold pressor pain	The area under pain curve % was highest in low-dose opioid and lowest in high-dose opioid with metamizole coming in between. Pain tolerance (in seconds) was highest in those taking high-dose opioids and lowest in those taking low-dose opioids with metamizole being intermediate once again
Metamizole/dipyrone for the relief of cancer pain: A systematic review and evidence-based recommendations for clinical practice [66]	Assessing cancer pain relief	Even in lower doses, metamizole decreased pain intensity significantly when compared to morphine. Higher doses were even more potent and were equally effective as 60 mg of morphine.
Metamizole/dipyrone for the relief of cancer pain: A systematic review and evidence-based recommendations for clinical practice [66]	Assessing acute postoperative pain relief after septoplasty	At 24 h postoperatively, VAS pain scores of metamizole were lowest at 18.1 +/− 11.9

**Table 3:** Overview of comparative investigations.

Study Title	Efficacy	Onset of Action	Duration	Side Effects
Treatment of postoperative pain after total hip arthroplasty: comparison between metamizol and paracetamol as adjunctive to opioid analgesics-prospective, double-blind, randomized study [56]	Metamizole demonstrated a significant reduction in pain AUC values (17.9) compared to paracetamol (30.6). It is cost-effective, being 10 times cheaper than i.v. paracetamol. Safety profile favours paracetamol.	Effective analgesia during the first 24 h	Not specified	Paracetamol has a better safety profile than Metamizole
Efficacy of intravenous paracetamol, metamizol and lornoxicam on postoperative pain and morphine consumption after lumbar disc surgery [53]	Metamizole showed significant pain reduction post-lumbar disc surgery compared to lornoxicam. Morphine consumption was higher in the metamizole group. Agranulocytosis risk is a concern for metamizole.	Onset is quicker in the metamizole group	Lower VAS pain scores at the end seen in those taking metamizole	Paracetamol is safer and preferred for supplemental analgesia after lumbar disc surgery
Tilidine and dipyrone (metamizole) in cold pressor pain: A pooled analysis of efficacy, tolerability, and safety in healthy volunteers [61]	Higher doses of tilidine showed slightly greater pain reduction than metamizole. Tilidine, however, caused side effects like dizziness, nausea, and vomiting.	Not specified	Not specified	Metamizole had lower side effects compared to tilidine
Efficacy of intravenous paracetamol compared to dipyrone and parecoxib for postoperative pain management after minor-to-intermediate surgery: a randomized, double-blind trial [56]	Metamizole demonstrated a greater decrease in VAS pain scores postoperatively. Patients required fewer rescue medications, but efficacy equalised with placebo after 1 week. Parecoxib group under review for safety concerns.	Quicker pain relief in metamizole group	At the end lower pain scores in metamizole	Metamizole has a longer duration of action and fewer rescue medication requests compared to paracetamol and parecoxib
Efficacy and tolerance of oral dipyrone versus oral morphine for cancer pain [60]	2 g metamizole efficacy comparable to morphine, with fewer side effects.	Metamizole 2 g showed quicker onset	Not mentioned; however, at day 7, pain scores were least for metamizole.	Incidence of side effects lesser in metamizole group
Metamizole/dipyrone for the relief of cancer pain: A systematic review and evidence-based recommendations for clinical practice [66]	The number needed to treat for achieving a 50% reduction in pain after minor surgeries was 2.1 for metamizole 500 mg, showcasing its superior efficacy compared to other drugs	Not mentioned	Not mentioned	Most number of side effects reported in those taking morphine
Comparison of cizolirtine citrate and metamizol sodium in the treatment of adult acute renal colic: a randomized, double-blind, clinical pilot study [51]	No statistical difference in pain reduction between cizolirtine citrate and metamizole, but non-inferiority of cizolirtine citrate not established. Cizolirtine citrate is associated with more adverse effects.	Early onset in metamizole group	Not statistically different. Comparable	Metamizole has comparable efficacy with fewer adverse effects
Postoperative analgesia at home after ambulatory hand surgery: a controlled comparison of tramadol, metamizol, and paracetamol [55]	Metamizole is rated as “adequate” in treating pain by more patients than tramadol and paracetamol. Tramadol caused more adverse effects, including nausea and tiredness.	Onset faster in tramadol followed by metamizole followed by paracetamol	Action of pain relief longest and VAS scores least in metamizole users	Metamizole demonstrated higher efficacy with fewer adverse effects compared to tramadol and paracetamol
Comparison of the analgesic efficacy of metamizole and tramadol in experimental pain [65]	Tramadol had a longer duration of pain relief, but with adverse effects such as dry mouth, nausea with vomiting, and sleepiness.	Faster onset of action in tramadol	Longer duration of action exhibited by tramadol	Metamizole had a shorter duration of action but fewer adverse effects compared to tramadol
Comparison of intravenous dexketoprofen and dipyrone in acute renal colic [52]	Dexketoprofen showed greater and faster analgesic effects compared to 2 g metamizole. Adverse effects were similar in renal and gastrointestinal systems for both drugs.	Faster onset of action by dexketoprofen	Time taken for patients to ask additional analgesics highest in metamizole	Similar adverse effects in both groups
Efficacy of lornoxicam for acute postoperative pain relief after septoplasty: a comparison with diclofenac, ketoprofen, and dipyrone [88]	Diclofenac and ketoprofen showed greater pain reduction compared to lornoxicam and metamizole, except at specific time points. Fewer patients needed additional opioids with diclofenac and ketoprofen.	Earlier onset shown by Lornoxicam followed by metamizole followed by diclofenac measured at 2 h postoperatively	Not mentioned. However, at 24 h, VAS scores were lowest in metamizole.	Metamizole is less effective compared to diclofenac and ketoprofen in reducing pain
Metamizole vs. ibuprofen at home after day case surgery: A double-blind randomized controlled noninferiority trial [58]	Metamizole and paracetamol showed a greater reduction in PACU compared to ibuprofen and paracetamol. Metamizole and paracetamol had a lower I.V. piritramide consumption. Different side effects observed in both groups.	Early onset in ibuprofen	Longer duration in ibuprofen. However, a need for rescue medication is less in metamizole.	Metamizole and paracetamol combination demonstrated higher efficacy with different side effects compared to ibuprofen and paracetamol
Analgesic Medication in Fibromyalgia Patients: A Cross-Sectional Study [43]	Reduction in pain severity was achieved better in those taking metamizole and NSAIDs	Higher number of patients took NSAIDs as an on-demand drug	Not mentioned	Greater number of patients withdrew NSAIDs due to no effect of the drug. However, side effects appeared greater in those taking metamizole.
Dipyrone is the preferred nonopioid analgesic for the treatment of acute and chronic pain. A survey of clinical practice in German-speaking countries [46]	Not mentioned	Not mentioned	Not mentioned	Change in blood values, symptoms of influenza, infection needing treatment, and treatment in the intensive care unit was observed in those taking metamizole. Fatal outcomes were reported by 11 physicians.
Use of tramadol or other analgesics in patients treated in the emergency department as a risk factor for opioid use [47]	Efficacy was similar in those taking NSAIDs and metamizole	Metamizole has a faster onset of action	Metamizole had a longer duration of action	Those taking tramadol were at risk of receiving opioids within 12 months leading to adverse effects caused by opioids
Efficacy and tolerance of metamizole versus morphine for acute pancreatitis pain [48]	Efficacy of metamizole is greater than morphine	Metamizole showed a non-significant quicker onset of analgesia	Not mentioned	Similar in both groups. 3 cases of vomiting and 1 case of somnolence in the morphine group and 1 case of vomiting in the metamizole group.
Parenteral dipyrone versus diclofenac and placebo in patients with acute lumbago or sciatic pain: randomized observer-blind multicenter study [49]	Greater reduction in pain scores in those taking metamizole	Faster onset in those taking metamizole	Not mentioned	Comparatively more adverse effects were seen in those taking metamizole.
Comparison of the onset and duration of the analgesic effect of dipyrone, 1 or 2 g, by the intramuscular or intravenous route, in acute renal colic [50]	Marked increase in efficacy were seen in those taking metamizole 2 g either by i.m or i.v	Faster onset seen in those taking metamizole 2 g i.v	Not mentioned	There were no significant differences in the appearance of adverse effects. Drowsiness was seen in those taking metamizole and dry mouth and hypotension was seen in those taking diclofenac sodium.
A randomized double-blind placebo-controlled study of dipyrone and aspirin in post-operative orthopaedic patients [54]	Relief scores were greatest in those taking metamizole	Onset was faster in those taking metamizole	Not mentioned	Greater number of side effects was seen in those taking aspirin in the form of burning in the abdomen, abdominal discomfort, nausea and vomiting, and itching
Oral metamizole (1 g and 2 g) versus ibuprofen and placebo in the treatment of lower third molar surgery pain: randomised double-blind multi-centre study. Cooperative Study Group [63]	Greater relief was seen in those taking metamizole 2 g	Onset of action was quicker in those taking metamizole	Not mentioned	Slight decrease from baseline blood pressure levels were seen in all groups. Moderate sleepiness occurred in those taking metamizole. Syncope associated with dizziness occurred in those taking ibuprofen and local bleeding occurred in those taking placebo.

## Data Availability

Not applicable.
